# The HSP90 inhibitor ganetespib: A potential effective agent for Acute Myeloid Leukemia in combination with cytarabine

**DOI:** 10.1016/j.leukres.2015.03.016

**Published:** 2015-06

**Authors:** M. Lazenby, R. Hills, A.K. Burnett, J. Zabkiewicz

**Affiliations:** Cardiff Experimental Cancer Medicine Centre (ECMC), Department of Haematology, School of Medicine, Cardiff University, Heath Park, Cardiff CF14 4XN, UK

**Keywords:** HSP90, AML, Ganetespib

## Abstract

HSP90 is a multi-client chaperone involved in regulating a large array of cellular processes and is commonly overexpressed in many different cancer types including hematological malignancies. Inhibition of HSP90 holds promise for targeting multiple molecular abnormalities and is therefore an attractive target for heterogeneous malignancies such as Acute Myeloid Leukemia (AML).

Ganetespib is a highly potent second generation HSP90 inhibitor which we show is significantly more effective against primary AML blasts at nanomolar concentrations when compared with cytarabine (*p* < 0.001). Dose dependant cytotoxicity was observed with an apoptotic response coordinate with the loss of pro-survival signaling through the client protein AKT. Combination treatment of primary blasts with ganetespib and cytarabine showed good synergistic interaction (combination index (CI): 0.47) across a range of drug effects with associated reduction in HSP70 feedback and AKT signaling levels.

In summary, we show ganetespib to have high activity in primary AMLs as a monotherapy and a synergistic relationship with cytarabine when combined. The combination of cytotoxic cell death, suppression of cytoprotective/drug resistance mechanisms such as AKT and reduced clinical toxicity compared to other HSP90 inhibitors provide strong rationale for the clinical assessment of ganetespib in AML.

## Highlights

• Ganetespib is a highly potent HSP90 inhibitor in primary AML blasts.
• Apoptotic induction is co-ordinate with suppression of pro-survival protein AKT.
• Synergistic interaction with AraC suppressing pro-survival targets HSP70 and AKT.
• Provides strong rationale for further clinical assessment of ganetespib in AML.

## 1 Introduction

Heat shock protein 90 (HSP90) is a key member of the heat shock protein family which act as molecular chaperones, facilitating protein folding and activation of client proteins that cover a diverse range of cellular functions including signal transduction *via* protein kinases, chromatin/epigenetic remodeling, vesicular transport, immune response, steroid signaling and regulation of viral infections [1–3]. HSP90 is abundantly expressed in eukaryotic cells with both constitutive and stress induced isoforms [4] and is often associated in complex with HSP70 and co-chaperones such as HSP40 and Cdc37 [5], which aid in client protein binding, ATP mediated activation and protection from proteosome degradation [6,7].

HSP90 overexpression has been reported in several malignancies [8–10] including hematological malignancies such as AML where overexpression has been linked with poor prognosis [3,11,12]. HSP90 acts as a chaperone to a large number of client proteins including SRC, KIT, RAL, JAK, AKT, ERBB2 and CDKs, many of which are oncogenically activated in cancer cells [13]. Drug resistance, cell survival and tumor progression may be critically dependent upon HSP90 function through the chaperones ability to protect mutant and oncogenic proteins from degradation. Given the molecular heterogeneity of AML, HSP90 inhibition could represent a logical therapeutic strategy.

Initial targeting of HSP90 focused on geldanamycin, a large naturally occurring compound and its ansamycin derivatives 17-AAG and 17-DMAG which mimicked the ATP binding site of HSP90 [14]. Therapeutic activity was observed in many malignancies [13], however poor pharmacological properties and toxicities limited their further progress [15]. Ganetespib belongs to the resorcinol group of second generation synthetic HSP90 inhibitors which are considerably smaller and work by competitively binding the N-terminal ATP binding site. Pre-clinical studies have shown ganetespib to have greater potency than first generation inhibitors such as 17-AAG in several cancers [16–18], including hematological malignancies [19]. It has also been shown to also overcome tyrosine kinase inhibitor (TKI) resistance [18]. Clinically, ganetespib has shown a favorable safety profile without the dose-limiting liver or ocular toxicities associated with other Hsp90 inhibitors [20,21], and has shown encouraging activity in a Phase 2 NSCLC trial [22]. As a prelude to clinical studies we assessed the *in vitro* effects of ganetespib in AML cell lines and primary AML blasts both as a single agent and in combination with cytarabine.

## 2 Materials and methods

### 2.1 Samples and cell culture

Bone marrow and peripheral blood samples were collected from newly diagnosed AML patients entering the NCRI AML15, 16 and 17 trials with the patients’ informed consent using documentation approved by the Wales Multicentre Research Ethics Committee. The clinical characteristics of the 52 patients are shown in Table 1. Primary mononuclear cells were enriched by density gradient centrifugation with Histopaque (Sigma, Poole, UK) and further analyzed for blast (leukaemic cell) purity by CD45 staining and flow cytometry. AMLs with >70% blasts following gradient fractionation were cryopreserved and used for subsequent analysis. HL60 cells were maintained in RPMI media supplemented with 10% fetal bovine serum (FBS). MV411 cells and primary AML blasts were cultured in IMDM media supplemented with 10% FBS. All cultures were maintained at 37 °C in a 5% CO_2_ humidified atmosphere. Cell viability was measured by trypan blue exclusion on a Cellometer Vision (Peqlab Ltd., Fareham, UK).

### 2.2 Cell viability assays

*In vitro* cytotoxicity assays were performed in 96 well plates on cell lines and primary material using the CellTiter96^®^ Aqueous one solution cell proliferation assay(MTS) according to the manufacturer's instructions (Promega UK Ltd., Southampton, UK). Primary cells (1 × 10^5^/well) and cell lines (1 × 10^4^/well) were treated with serial dilution dose range of ganetespib or cytarabine (AraC) in triplicate and IC_50_ values calculated using Calcusyn software (Biosoft, Cambridge, UK).

Synergy between ganetespib and Ara-C was assessed in cell lines and primary AML samples using an experimentally determined fixed molar ratio of ganetespib with AraC within clinically relevant doses (1:100, 1:50, 1:10 ratios). Drugs were set up singly and in combination and Calcusyn software was used to determine combination index (CI) values according to the Chou and Talalay method [23]. CI values of <1 were considered synergistic.

### 2.3 Flow cytometric analysis of apoptosis

HL60 and NB4 AML cell lines were treated with ganetespib at concentrations between 10 and 250 nM, and cultured for 24 h, 48 h and 72 h. Annexin V positivity was measured using the Annexin V Apoptosis Detection Kit (eBioscience, Hatford, UK) according to the manufacturer's instructions. Briefly, cells were washed in phosphate buffered saline (PBS) and incubated with fluorescein-labeled Annexin V for 10 min. Cells were then washed and resuspended in 1 μg/ml propidium iodide (PI) prior to assessment by flow cytometry (Accuri Cytometers (Becton Dickinson, UK)). All experiments were performed in triplicate.

### 2.4 Immunoblotting

AML cells were treated with increasing doses of ganetespib for 48 h and washed 3 times in ice-cold phosphate-buffered saline (PBS) then lysed in 20 mM Tris [pH 7.4], 150 mM NaCl, 1% Igepal (Sigma–Aldrich, Poole, UK), 10% glycerol, 10 mM EDTA, 20 mM NaF, and 3 mM NaVO_4_ plus complete protease inhibitors (MiniComplete EDTA-free; Roche, Burgess Hill, UK) for 30 min at 4 °C followed by centrifugation at 16,000 × *g*. The clarified protein lysates were quantified and subjected to Western blotting as previously described [24] using antibodies for HSP90, AKT, IKBα (Cell Signaling Technology Inc., New England Biolabs, Hitchin, UK), HSP70 (Millipore (UK) Ltd., Watford, UK). Blots were reprobed for equal loading using GAPDH (Cell Signaling Technology Inc., New England Biolabs, Hitchin, UK) and quantified using AIDA image analysis software (Raytest UK Ltd.).

## 3 Results

### 3.1 Ganetespib shows high potency in primary AML samples

**Fig. 1:**
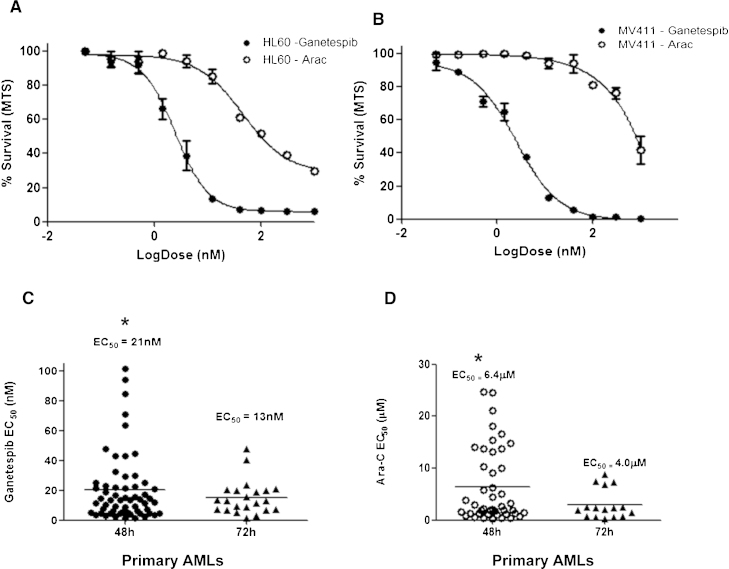
Ganetespib shows improved efficacy compared to AraC in AML. Dose response curves for (A) HL60 and (B) MV411 cells following drug exposure for 72 h measured by MTS cell proliferation assay (% survival calculated compared to equivalent vehicle control). (C) MTS ganetespib drug efficacy in primary AML cells at 48 (*n* = 62) and 72 h (*n* = 22). (D) AraC efficacy in primary cohort at 48 and 72 h. *Ganetespib *vs* AraC EC50 *p* < 0.0001 at 48 and 72 h. Ganetespib 48 *vs* 72 h *p* = 0.4.

The cytotoxicity of ganetespib was initially assessed by MTS assay using the myeloid cells lines MV411 and HL60. Analysis of ganetespib dose response curves showed increased efficacy compared with cytarabine, with low nanomolar EC50s of 8.4 nM and 2.7 nM for HL60 and MV411 respectively (Arac EC50; 116 nM and 961 nM respectively) at 72 h (Fig. 1A and B). The main aim of the project was to assess sensitivity in primary samples which have had various responses to conventional chemotherapy. Ganetespib activity at 48 h was then assessed in primary blasts isolated from diagnostic AML patients (*n* = 62). Ganetespib was significantly more potent than AraC in primary blasts with an EC50 of 20.9 ± 21 nM and 6.4 ± 7 μM respectively (*p* < 0.001, MWU, Fig. 1C and D). There was no significant improvement in ganetespib activity at 72 h (*p* = 0.4, MWU, *n* = 22) compared with a 48 h assessment, so further drug analysis was carried out using the 48 h time point.

To assess the clinical correlations associated with *in vitro* drug sensitivity, we studied the relationship between clinical outcome in patients treated with conventional intensive chemotherapy in relation to log_10_(EC_50_) of ganetespib (Patient and treatment details, Table 1). There was no significant association with patients characteristics, although there was a trend for higher EC_50_ levels to be associated with higher age. Survival data was available on 54/56 intensively treated patients. We found no significant association between EC_50_ and relapse risk or survival when analyzed with EC_50_ (OR 1.02 (0.20–5.30) per 10-fold increase in EC_50_, *p* = 1.0) or relapse free survival (HR 1.31 (0.55–3.10) per 10-fold increase in EC50, *p* = 0.6) or overall survival (HR 1.73 (0.84–3.55) per 10-fold increase in EC_50_, *p* = 0.13) in univariate analyses. In analyses adjusted for age, WBC, performance status, secondary disease and cytogenetics, however, there was some evidence that higher EC_50_ may be associated with a trend for worse survival although this did not reach significance (HR 2.53 (0.94–6.77) *p* = 0.07). This data builds a rationale for taking ganetespib into the clinic as it can potentially target cells from all AML patients equally, even those who did poorly with conventional chemotherapy.

**Fig. 2:**
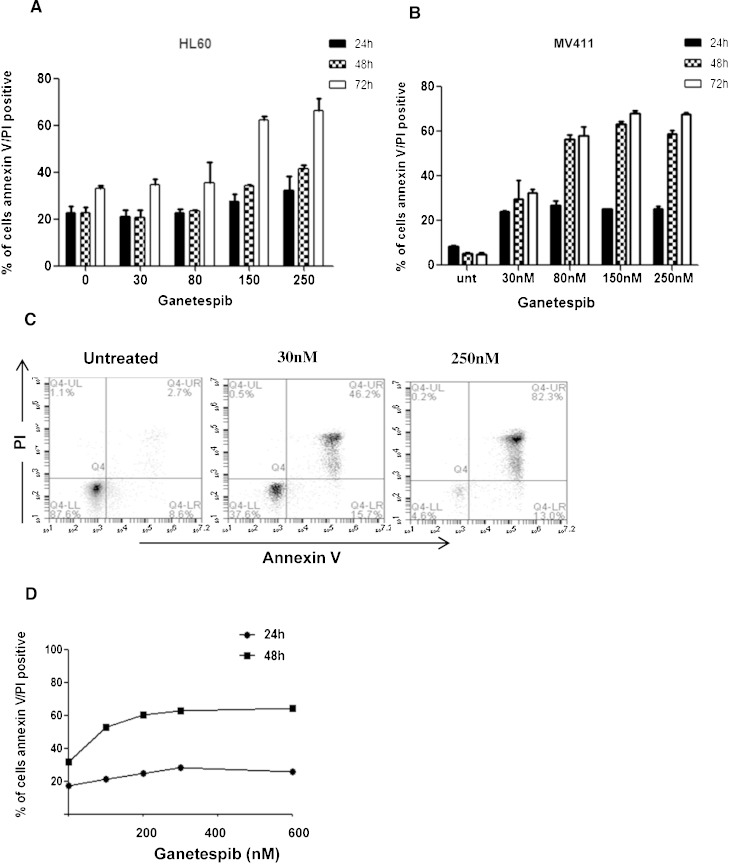
Ganetespib induces dose dependant induction of apoptosis. Apoptotic induction of Annexin V/PI incorporation in (A) HL60 and (B) MV411cell lines measured at 24, 48 and 72 h by flow cytometry. All experiments were performed in triplicate. (C) Example flow cytometry data of dual Annexin V/PI staining of MV411 cells at maximal response. (D) Primary cell AnnexinV/PI induction following ganetespib dosing over a 24 h and 48 h period.

Annexin V/PI incorporation was investigated by flow cytometry to confirm a cytotoxic method of action for ganetespib in AML over a time course of 24, 48 and 72 h. AML cells showed a dose and time dependant increase in apoptotic induction in response to ganetespib (Fig. 2A–C). This effect was also observed in primary AML cells (Fig. 2D).

### 3.2 HSP90 targeting with ganetespib results in client protein degradation

**Fig. 3:**
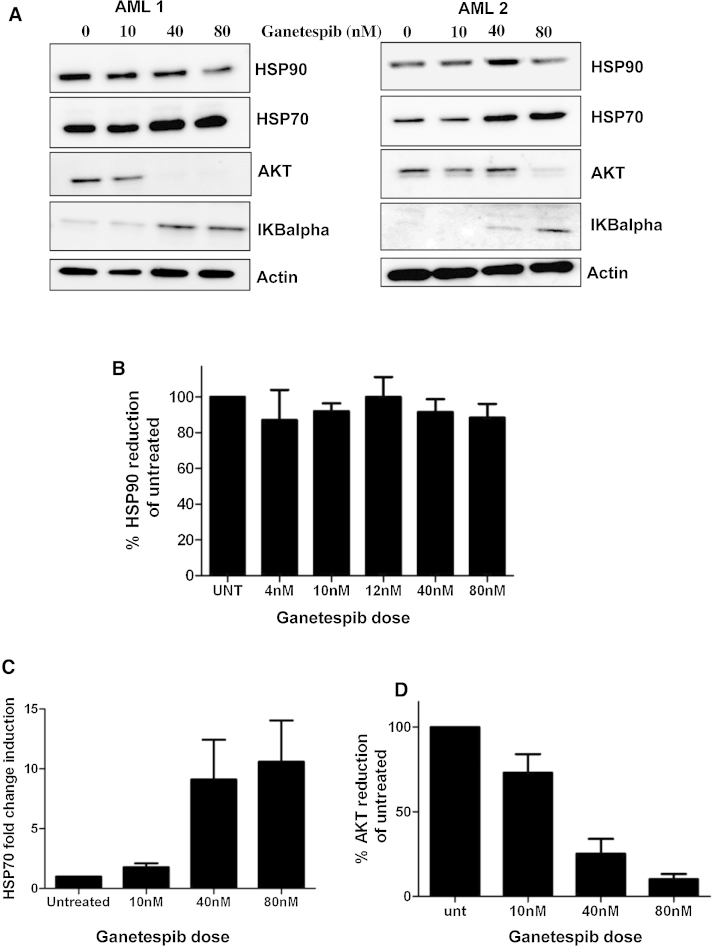
Primary AML blasts show client protein knockdown following ganetespib treatment. (A) Representative western blot of primary AML 48 h following ganetespib treatment. Quantification of (B) HSP90 *n* = 10, (C) HSP70 *n* = 6, (D) AKT *n* = 6, protein expression in response to increasing doses of ganetespib. All samples were normalized to GAPDH protein levels and expressed as a percentage or fold change relative to untreated samples.

Ganetespib treated AML blasts were subjected to Western blot analysis for HSP protein response and client protein knockdown (Fig. 3A). Although total levels of HSP90 protein were maintained following blast incubation with ganetespib (*n* = 10), a dose dependant increase in the chaperone protein HSP70 was seen at 48 h (Fig. 3B and C). Given that the use of HSP90 inhibitors may block pro-survival resistance mechanisms in AML blasts such as AKT overexpression, quantitation of AKT levels following ganetespib exposure was performed in a cohort of primary AML cells (*n* = 6) and results show a dose dependant loss of AKT expression (Fig. 3D).

### 3.3 Ganetespib shows synergistic action with cytarabine

**Fig. 4:**
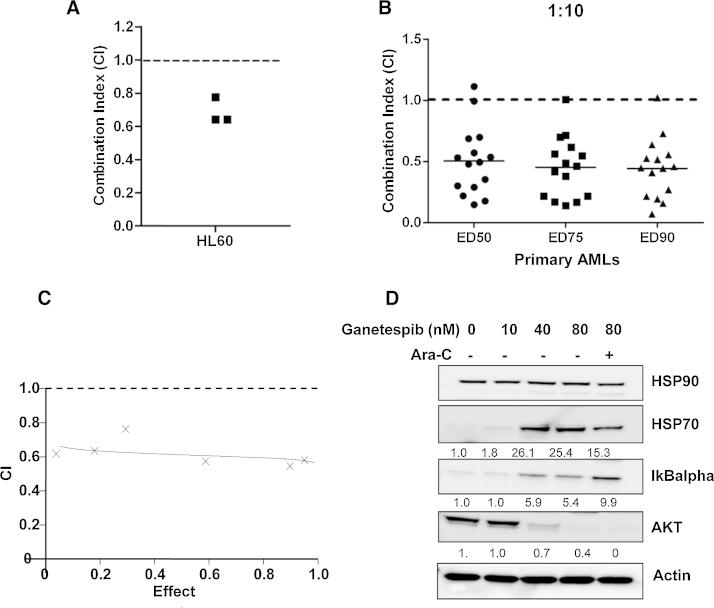
Ganetespib synergizes with AraC. (A) Combination index (CI) values were calculated between ganetespib and AraC in myeloid cell line HL60 at a 1:250 molar ratio (B) CI values for primary AML samples (*n* = 15) at 1:10 ratio (ganetespib:AraC). A CI value of <1 is indicative of synergy between the agents. (C) Representative data showing CI range in response to increased drug effect (D) representative Western blot of primary AML showing ganetespib target effects alone and in combination with AraC. Densitometry analysis of fold change relative to untreated control is expressed below each panel.

Cytosine arabinoside is a standard agent for leukemia and produces remission rates of 25–80% when used in a high-dose intermittent schedule in AML relapse. Given that DNA repair proteins can lessen the activity of cytarabine and several of these factors are HSP90 client proteins [25], we assessed whether ganetespib could enhance the activity of cytarabine in AML blasts and AML cell lines. Synergistic ratios between the two agents were first assessed in AML cell line HL60 (Fig. 4A) using serial dilutions of fixed molar ratios and then in a cohort of primary AML blasts (*n* = 15). As the efficacy of cytarabine in primary blasts is reduced, ratios of 1:10, 1:50 and 1:100 were investigated. Combination index (CI) values were established for each patient and ganetespib showed good synergy with cytarabine (CI ave = 0.47) at a 1:10 dose ratio across a range of dose effects (Fig. 4B–D). Western blot analysis of combination treated AML blasts showed a small increase in client protein AKT knockdown and also interestingly, up regulation of IKBα (a repressor of the pro-survival protein NFκB known to confer drug resistance in AMLs [26]) and reduced HSP70 induction in response to combination treatment compared to an equivalent dose of ganetespib alone.

## 4 Discussion

This study demonstrates that the novel HSP90 inhibitor, ganetespib, is an effective agent against primary AML blasts at nanomolar concentrations which are clinically achievable [21] and far superior to the standard agent, cytarabine. Previous studies of HSP90 inhibition have shown similar anti-proliferative effects in AML and other leukaemias [27–30], although ganetespib exhibits considerably greater potency than has been reported with previous HSP90 agents in primary AML samples [3,30]. The clinical development of many HSP90 inhibitors has been limited by toxicities, particularly ocular toxicity [13,29], but the clinical development of ganetespib to date suggests that the drug is well tolerated and that the ocular toxicity is infrequent, in contrast to some other second generation HSP90 inhibitors [16,20,21].

Induction of dose dependent apoptosis was observed in AML cells indicating a cytotoxic method of cell death in response to treatment. Annexin induction of cell death occurred at slightly higher drug doses than observed for the MTS assay and this may be partially due to the action of ganetespib on cell cycle regulator clients of HSP90. Ganetespib has already been shown to induce growth arrest and apoptosis in several other cancer models [18,31,32].

Although total HSP90 protein levels remained unchanged by HSP90 inhibition (in line with previous reports [33]), we demonstrated client protein knockdown at nanomolar doses of the pro-survival kinase AKT, which has been previously reported to mediate drug resistance and poor prognosis in AML [34]. AKT is just one of a number of client proteins (known or unknown) for HSP90 that may be targeted by ganetespib treatment and knockdown of multiple HSP90 clients such as KIT, Ral, JAK2 and members of the CDK family [5] may contribute to the observed high efficacy of this drug in primary AML samples. Concurrently we observed upregulation (although transient) of the chaperone HSP70 by ganetespib. This upregulation of HSP70 by HPS90 inhibitors has been reported as a cytoprotective function in response to HSP90 inhibition with sustained induction of the HSP transcription factor HSF1 driving a potential feedback mechanism by which other HSPs are also upregulated [6]. Induction of HSP70 has been reported to lead to drug resistance and poor prognosis in several cancer types [35–38] including AML [9]. It has also been previously used as a readout of HSP90 inhibitor action in the clinic [11,39], including initial ganetespib studies [21]. Knockdown of HSP70 using pharmacological inhibitors increases the efficacy of HSP90 inhibition in AML [40], however several time course studies report HSP70 upregulation as transient and diminishing with disease progression and may not predict patient outcome [6,9], suggesting a limited role for HSP70 as a biomarker of response.

Previous reports show HSP90 knockdown can sensitize cells to DNA damage inducing agents [41] providing good rationale for combination therapy. However, as HSP90 inhibition can cause cell cycle arrest, there may be concerns about combination with S-Phase inhibitors such as cytarabine. Our pre-clinical data suggest ganetespib and cytarabine combination shows good synergistic interaction when co-administered *in vitro* at a range of clinically relevant doses including those used in the recent Li-1 trial. This data is in line with previous combination studies in myeloma cells where co-administration rather than sequential dosing of agents giving maximum synergistic effects [36]. Our combination data also shows reduced HSP70 induction compared to ganetespib alone, reducing the possible resistance issues associated with induction of this chaperone. This supports the rationale for clinical development of ganetespib in combination with the standard cytarabine therapy as has been initiated in ISRCTN40571019.

Given the redundancy of many protein kinases in tumor maintenance, the effectiveness of any inhibitor may rely on the oncogene addiction to the HSP90/client protein [42]. The multi-client action of HSP90 affords ganetespib the ability to inhibit many more targets than typical kinase inhibitors, and in combination with other chemotherapeutic and novel agents will allow ganetespib maximum targeting of diverse molecular abnormalities such as those found in AML.

## Authorship and disclosures

JZ/AKB were the principle investigators; ML performed the laboratory work for this study; JZ co-ordinated the research; JZ and ML wrote the paper. RKH performed clinical correlation analysis. The authors declare that there are no competing financial interests in relation to the work described.

## Conflict of interest statement

The authors have no conflicts of interest to declare.

## Figures and Tables

**Table 1:** Patient characteristics.

Characteristic	Number (%)	Median EC_50_ (nM) and range	*p*-Value (relation between EC50 and characteristic)
**All data**		21.6 (1.4–272.9)	

**Trial**^e^			0.6^b^
AML15	21 (34%)	15.3 (4.5–106.8)	
AML16	9 (15%)	22.6 (2.9–101.7)	
AML17	30 (48%)	21.9 (1.4–272.9)	
AML LI-1	2 (3%)	47.2 (27.3–67.2)	

**Treatment regimen**			0.2^b^
Intensive chemo	56 (90%)	21.1 (1.4–272.9)	
Non-intensive	6 (10%)	45.4 (98.9–84.6)	

**Age (years)**			0.06^c^
0–29	6 (10%)	17.1 (3.5–70.9)	
30–39	7 (11%)	21.7 (4.5–58.3)	
40–49	12 (19%)	14.9 (1.4–272.9)	
50–59	4 (26%)	22.0 (5.6–106.8)	
60+	21 (34%)	22.9 (2.9–116.2)	
Median (range)	54 (0–88)		

**Sex**			0.9^b^
Male	27 (44%)	21.7 (2.9–272.9)	
Female	35 (56%)	21.4 (1.4–106.8)	

**WBC**			0.5^c^
0–9.9	2 (3%)	13.9 (6.4–21.4)	
10–49.9	28 (45%)	23.6 (2.9–101.7)	
50–99.9	15 (24%)	44.6 (1.4–272.9)	
100+	17 (27%)	15.3 (4.0–44.7)	
Unknown			
Median (range)	55.0 (7.5–249.0)		

**Type of AML**			0.13^b^; 0.2^d^
De Novo	54 (87%)	21. 6 (1.4–272.9)	
Secondary	7 (11%)	50.8 (4.5–116.2)	
MDS	1 (2%)	2.9	

**FAB group**			0.3^b^
M0	0		
M1	7 (16%)	21.7 (6.4–66.0)	
M2	6 (13%)	37.9 (11.8–101.7)	
M4	17 (38%)	21.4 (1.4–272.9)	
M5	15 (33%)	14.9 (3.5–116.2)	
Unknown/other	17		

**Cytogenetic group**			0.07^c^
Favorable	4 (8%)	52.8 (4.5–272.9)	
Intermediate	46 (88%)	19.8 (1.4–116.2)	
Adverse	2 (4%)	9.5 (4.0–14.9)	
unknown	10		

**WHO performance status**^a^			0.8^c^
0	31 (51%)	21.4 (2.9–272.9)	
1	25 (41%)	25.1 (4.5–116.24)	
2	2 (3%)	34.3 (1.4–67.2)	
3	3 (5%)	20.7 (15.3–21.7)	

**FLT3 status**			0.9^b^
ITD wt	35 (58%)	22.9 (2.9–272.9)	
ITD mutant	25 (42%)	20.7 (1.4–116.2)	
ITD unknown	2		

TKD wt	52 (90%)		0.7^b^
TKD mutant	6 (10%)	21.1 (1.4–272.9)	
TKD unknown	4	24.7 (13.5–58.3)	

**NPM1 status**			0.3^b^
WT	31 (53%)	17.3 (2.9–272.9)	
Mutant	28 (47%)	21.9 (1.4–106.8)	
Unknown	3		

a One pediatric patient did not complete the WHO performance status cscale and instead completed the play performance scale.

b Wilcoxon Rank-Sum/Kruskal–Wallis test for difference between groups.

c Spearman correlation coefficient for continuous data/ordered groups.

d Test for secondary *vs* not secondary disease.

e Trials AML15, 16 and 17 patients were treated intensively (2 rounds of either: ADE (daunorubicin, cytarabine, etoposide), DA/DAT (daunorubicin, cytarabine/daunorubicin, cytarabine, thioguanine), FLAG-Ida (fludarabine, cytarabine, idarubicin, G-CSF) followed by two rounds of consolidation/novel agents,follow-up complete to 1/1/2014). AML16 non-intensive and LI-1 received low dose cytarabine based therapy. Apoptotic response in cell lines and primary samples.
